# Validity and reliability of two brief physical activity questionnaires among Spanish-speaking individuals of Mexican descent

**DOI:** 10.1186/1756-0500-7-29

**Published:** 2014-01-13

**Authors:** Sonia Vega-López, Adrian Chavez, Kristin J Farr, Barbara E Ainsworth

**Affiliations:** 1 School of Nutrition and Health Promotion, Arizona State University, 500 North 3rd Street, Phoenix, AZ 85004, USA

**Keywords:** Physical activity assessment, Validity, Reliability, Survey, Spanish

## Abstract

**Background:**

Mexican Americans are the largest minority group in the US and suffer disproportionate rates of diseases related to the lack of physical activity (PA). Since many of these Mexican Americans are Spanish-speaking, it is important to validate a Spanish language physical activity assessment tool that can be used in epidemiology as well as clinical practice. This study explored the utility of two Spanish translated physical activity questionnaires, the Stanford Brief Activity Survey (SBAS) and the Rapid Assessment of Physical Activity (RAPA), for use among Spanish-speaking Mexican Americans.

**Methods:**

Thirty-four participants (13 M, 21 F; 37.6 ± 9.5 y) completed each of the two PA surveys twice, one week apart. During that week 31 participants also wore an *ActiGraph* GT1M accelerometer for 7 days to objectively measure PA. Minutes of moderate and vigorous PA (MVPA) were determined from the accelerometer data using Freedson and Matthews cut points.

**Results:**

Validity, determined by Spearman correlation coefficients between questionnaire scores and minutes of *ActiGraph* measured MVPA were 0.38 and 0.45 for the SBAS and RAPA, respectively. Test-retest reliability was 0.61 for the SBAS and 0.65 for the RAPA. Sensitivity and specificity were 0.60 and 0.47 for the SBAS, and 0.73 and 0.75 for the RAPA. Participants who were classified as meeting the 2008 National Physical Activity Guidelines by the RAPA engaged in significantly (p < 0.05) more minutes of MVPA than those who were not, while there were no significant differences in minutes of MVPA classified by the SBAS.

**Conclusions:**

The SBAS and the RAPA are both reasonably valid measures for quickly assessing PA and determining compliance to the PA guidelines in Spanish-speaking Mexican Americans. Although the two questionnaires had comparable reliability, the RAPA was better able to distinguish between those who met and did not meet National PA Guidelines.

## Background

Hispanics are the largest ethnic minority group in the United States constituting 16% of the population, of which 63% are of Mexican descent [1]. Relative to other ethnic groups, Mexican Americans are considered to be at increased risk for the development of chronic diseases. This is in part due to disparities in the prevalence of risk factors such as obesity [2-4], cardiovascular disease risk factors [5], inflammatory markers [6,7], insulin resistance [8], and metabolic syndrome and its components [9]. Moreover, Mexican Americans have been reported to have a higher prevalence of diabetes than non-Hispanic blacks or whites [10,11]. It is well established that risk factors for chronic conditions can be improved by regular physical activity (PA) [12]. In turn, low levels of PA are associated with an increased risk of mortality [13,14] and risk for diabetes and cardiovascular disease [15,16].

Current National PA Guidelines recommend at least 150 minutes of moderate intensity PA weekly for the adult population [17]. Recent estimates indicate that many Americans are not meeting these recommendations. According to 2008 data from the National Health Interview Survey, 43.5% of all American adults met the 2008 PA guidelines; among Hispanics, only 33.4% met these recommendations [18]. In most observational studies, physical activity and physical fitness are lower among Mexican Americans relative to African Americans and non-Hispanic whites [19,20].

Questionnaires are commonly used to assess PA in epidemiological research as well as clinical practice. Compared to alternative methods of assessing PA, questionnaires are short, easy to administer, and require minimal resources, making them ideal for administration to large populations or as a quick screening tool [21]. A potential disadvantage is that surveys often times focus on leisure-time PA and do not assess occupational PA. This may limit the utility of many surveys for use in a Mexican population, since previous research has shown that Mexicans, have high rates of occupational PA relative to other ethnic groups [22,23]. In a sample of 76,820 adults living in the US obtained from the NHIS (2000-2003), 39% of Mexicans and 19% of Mexican Americans reported having a physically active occupation compared to only 12% of non-Latino whites [22]. Therefore, assessing only leisure-time PA may underestimate true PA participation in this population.

Existing PA instruments validated for use with Spanish speakers [24-27] are relatively lengthy and may not be ideal for population-based research or clinical practice. For example, the Minnesota Leisure-Time PA Questionnaire (68-78 items) and the Seven-day Physical Activity Recall have been translated into Spanish [24,28], but both are interviewer administered and may take as long as 20 minutes to complete. Others rely on numeral literacy in completing the answers. For example, the International Physical Activity Questionnaire [26] is relatively short with 7 items, but requires complex mathematical calculations in recalling the number of days in a past week and then multiplying the days of activity by the average hours and minutes of participation for all of those days. Such mathematical calculations are difficult and are subject to reporting errors. In addition, these questionnaires have not been tailored for specific sub-groups of the Hispanic population of the United States. Language and cultural differences may affect comprehension and interpretation of a questionnaire when administered to a diverse population, compromising the validity of the responses [27,29]. Thus, assessing the validity of short and accurate PA surveys that can be used in the Spanish speaking Mexican-American population is warranted.

The purpose of this study was to assess the validity and reliability of two physical activity surveys, The *Stanford Brief Activity Survey* (SBAS) [30] and the Mexican Spanish version of the *Rapid Assessment of Physical Activity* (RAPA) [31], against accelerometer measured physical activity. These surveys were selected because they are both short and relatively easy to administer, making them ideal for use as brief physical activity screening tools. The RAPA has been previously translated into Mexican Spanish and is available for public use, but has not been validated with the target population. The SBAS is a unique questionnaire in that it assesses both occupational and leisure time physical activity in two questions. This questionnaire has been previously validated for use in an older English speaking population, but a Spanish language version of this questionnaire has not previously been developed or validated.

## Methods

### Participants

Thirty-seven Spanish-speaking self-reported Mexican or Mexican-American adults (15 males, 22 females; 21-56 years old) free of known chronic diseases were recruited from the Phoenix metropolitan area by distributing fliers, advertisements in Hispanic serving publications, and by word of mouth. Participants were excluded if they met any of the following criteria: (a) known chronic conditions (diabetes, cardiovascular, kidney or thyroid disease, cancer, etc.), (b) following a specific diet regimen (veganism, very low carbohydrate diet, etc.), (c) inability to walk for exercise, (d) pregnancy or breastfeeding, (e) use of lipid lowering or antihypertensive medications, and (f) participation in any other research study. These criteria were used to exclude individuals who may have unusual diet due to a medical condition or self-selected dietary restrictive habits. The study was approved by the Institutional Review Board at Arizona State University, and all participants provided written informed consent prior to being enrolled in the study.

### Study design

Participants attended two visits at the Nutrition Laboratory at Arizona State University. On the day of the first visit blood pressure and anthropometric measurements were taken, and a survey was administered in Spanish by a trained bilingual interviewer to collect PA (see below) and sociodemographic information. Participants were trained on the proper use of an ActiGraph GT1M accelerometer (ActiGraph, Pensacola, FL), and were instructed to wear it for 7 consecutive days starting the day after the first visit. A second visit was scheduled on the 8^th^ day to collect the accelerometer and re-administer the PA surveys in Spanish. When this day fell on a weekend, participants were instructed to delay the start day so that the 8^th^ day fell on Monday.

Data from three participants were excluded from the entire analysis for non-compliance with wearing the accelerometer (n = 2; see criteria below) and improper positioning of the accelerometer (n = 1). Therefore, thirty-four participants were included in analyses (13 males, 21 females). Data from one participant was excluded from the SBAS test re-test reliability calculations because the baseline SBAS was not completed. Accelerometer data were not available from three participants due to accelerometer malfunction; these individuals were excluded from the validity assessment analyses.

### Blood pressure and anthropometric measurements

Weight (in kilograms) and body composition (as % body fat) were measured using a Tanita body composition analyzer (Model TBF–300 A, Arlington Heights, IL). Height was measured in centimeters using a wall mounted stadiometer. Waist and hip circumferences were measured using a flexible tape measure at the umbilicus and at the largest portion of the hips, respectively. Blood pressure was taken following a 5 minute rest using an electronic sphygmomanometer (IntelliSense Blood Pressure Monitor HEM-907XL, Omron Healthcare, Kyoto City, Japan). All measurements were taken in triplicate and the mean of the three measurements was used for analyses. Body mass index (BMI) was calculated as mean weight in kilograms divided by mean height in meters squared (kg/m^2^).

### Physical activity assessment

*ActiGraph GT1M* accelerometers (ActiGraph LLC, Pensacola, FL) were used to objectively measure time spent in ambulatory PA’s of different intensities. The *ActiGraph* is a small, battery operated electronic motion solid state sensor (micro-electro-mechanical systems) designed to measure the rate and magnitude of body movement in a vertical plane (accelerations). Output data are digitized at a rate of thirty times per second with intensity data recorded in one minute epochs (sampling interval). The *ActiGraph* outputs data as counts per minute (cpm) that reflect: (a) the intensity of movement based on the frequency of acceleration deflections and (b) the duration of sustained period of the deflections. The *ActiGraph GT1M* has been validated as an accurate measure of energy expenditure when compared against the doubly labeled water method [32]. Intensity of activity was categorized based on cut-points developed from controlled laboratory experiments as follows: sedentary (<100 cpm) [33], light (100-759 cpm) and moderate-lifestyle activities (760-1951 cpm) [34], moderate-walking (1952-5725 cpm) and vigorous-intensity activities (>5725 cpm) [35]. The sum of minutes per day of moderate and vigorous PA (MVPA) over the seven days of wear time was calculated using these categories.

Participants were instructed to wear the ActiGraph over the right hip for seven days during all waking hours, only removing it to perform water-related activities (e.g., bathing, swimming). The ActiGraph was programmed to capture accelerations beginning at midnight of the day the instrument was provided to the participant. As determined previously by Matthews et al. [36], to characterize activity levels with at least 80% reliability participants needed to wear the ActiGraph continuously for 3-4 days to characterize moderate-and vigorous-intensity movements. Consecutive accelerometer counts of zero for 60 minutes or longer was considered non-wear time and removed from time spent as daily wear time. Thus, ≥4 days of data with counts recorded for ≥10 hours•d^-1^ were required for inclusion in the database. All participants included in the final study sample met this criteria and had 7 days of at least 10 hours of recorded wear time. Time spent at each physical activity intensity level is reported as the sum over these 7 days (min•wk^-1^).

Subjective measurement of PA was assessed using the SBAS [30] and the Mexican Spanish version of the RAPA [31] to estimate usual amount and intensity of PA performed by study participants. These questionnaires were both interviewer administered to ensure comprehension and account for potential variations in literacy levels.

The *Stanford Brief Activity Survey* (SBAS) is a brief, two item survey designed to assess occupational and leisure-time PA levels without relying on the recall of time spent in various types of activity [30]. This two-item questionnaire separately classifies the level of occupational and leisure-time PA performed by an individual. Each item provides examples of activities with increasing degree of intensity ranging from sedentary to high intensity activities. The SBAS rates occupational and leisure-time PA on a 5-point scale corresponding to classifications as inactive, light intensity, moderate intensity, hard intensity, and very hard intensity (rated as 1 through 5, respectively). Validation studies for an English language SBAS showed a strong, dose–response relation with minutes of moderate-intensity PA and energy expenditure (*p* < 0.01), and with cardiovascular disease risk factors (fasting glucose and insulin, triglycerides, HDL-C; *p* < 0.01) [30], and test-retest reliability is r = 0.62 l (*p* < 0.001) [37]. Prior to use, the SBAS was translated into Mexican Spanish by a bilingual/bicultural researcher (SV-L) and subsequently back translated by another bilingual research staff member who was not affiliated with the research team as previously recommended [38]. The Spanish version was a verbatim translation from the English version, with appropriate adjustments for accuracy and grammar. The purpose was not to introduce cultural adaptations (e.g. changing examples of activities to more culturally-relevant ones) so that the original version of the SBAS could be used.

The *Rapid Assessment of Physical Activity* (RAPA) is a 9-item, self-administered questionnaire readable at the sixth grade reading level, designed to assess current levels of leisure-time PA in the clinical setting [31]. The first seven items capture level and intensity of leisure-time PA based on pictorial examples of a variety of light, moderate, and vigorous activities; the last two items assess strength and flexibility training. The total score of the first seven items capture the PA level in a 5-point rating as sedentary, underactive, regular underactive/light, regular underactive, and regular active (rated as 1 through 5, respectively). In a validation study among older adults [31] the RAPA showed a positive correlation with PA level on the PA surveys from the Community Healthy Activities Model Program for Seniors (CHAMPS; r = 0.48; p < 0.001) [39], the Patient-centered Assessment and Counseling for Exercise (PACE; r = 0.56; p < 0.001) [40], and the Behavioral Risk Factor Surveillance System (BRFSS; r = 0.59; p < 0.001). The publicly available Mexican Spanish version of the RAPA was used for this study (available from http://depts.washington.edu/hprc/rapa, accessed on 5/20/2013).

### Statistical analysis

All statistical analyses were carried out using the IBM Statistical Program for the Social Sciences (SPSS) 19.0 for Windows (SPSS, Inc., Chicago, IL). Data are presented in text and tables as mean ± standard deviation (SD). Differences were considered significant at the 0.05 alpha level.

All comparisons between accelerometer measured PA and the questionnaire-assessed PA were made using data from the first administration of the questionnaires. Criterion validity of the two questionnaires was assessed using Spearman’s rank ordered correlation coefficients between the score on each of the questionnaires and the minutes of MVPA. One week test-retest reliability was determined using Intraclass Correlation Coefficients (ICC).

Sensitivity, specificity, and positive and negative predictive values were calculated by hand using accelerometer measured PA as the criterion to determine the ability of each questionnaire to predict whether or not a participant is meeting the 2008 U.S. PA guidelines [41]. Prior to conducting these analyses, data were dichotomized using the following criteria to classify participants as meeting the 2008 U.S. PA guidelines: (1) if accelerometer data indicated ≥150 min•wk^-1^ of MVPA; (2) if SBAS score was 3 or higher (moderate, hard, or very hard level of activity) [30]; or (3) if RAPA score was 5 (regular active) [31]. T-tests were performed to determine if there were differences in MVPA among those who were classified as meeting and not meeting PA guidelines for each of the two questionnaires.

## Results

Thirty-four (13 M, 21 F) participants completed all or part of this study (Table 1). A majority of participants were female (62%), bilingual in English and Spanish (88%), and were born in the U.S. (56%). Mean residence time in the U.S. was 28.4 ± 16.0 y. Participants’ mean BMI was 29.1 ± 5.7 kg/m^2^; 26% of participants were classified as being overweight and 44% of participants were classified as being obese. All participants had normal blood pressure (systolic blood pressure = 114.4 ± 12.1 mm Hg; diastolic blood pressure = 72.3 ± 9.2 mm Hg).

**Table 1:** Descriptive characteristics of study participants

**Characteristic**	**n (%)**	**Mean ± SD (n = 34)**	**Median (Interquartile range)**
Gender			
Male	13 (38)		
Female	21 (62)		
Language			
Bilingual	30 (88)		
Spanish only	4 (12)		
Place of birth			
U.S.	19 (56)		
Outside U.S.	15 (44)		
Monthly household income			
< $1000	8 (23.5)		
$1000-$2000	7 (20.6)		
$2000-$3000	7 (20.6)		
$3000-$4000	4 (11.8)		
> $4000	7 (20.6)		
Education (highest level completed)			
Elementary or middle school	4 (11.8)		
High school	20 (58.8)		
College	10 (29.4)		
Time in U.S. (y)		28.4 ± 16.0	30.0 (31.0)
Age (y)		37.6 ± 9.5	38.5 (16.0)
Weight (kg)		79.9 ± 17.9	77.2 (26)
Body mass index (kg/m^2^)		29.1 ± 5.7	29.2 (7.2)
Waist circumference (cm)		96.5 ± 14.2	95.3 (18.0)
Hip circumference (cm)		108.9 ± 11.7	107.8 (15.0)
Body fat (%)		34.1 ± 9.2	34.1 (13.8)
Systolic blood pressure (mm Hg)		114.4 ± 12.1	112.8 (17.0)
Diastolic blood pressure (mm Hg)		72.3 ± 9.2	73.5 (12.0)
MVPA (min•wk^-1^)		153.3 ± 88.8	140.0 (105.0)
Meeting U.S. PA guidelines^1^	15 (48)		

^1^Based on accelerometer MVPA.

### Participants’ physical activity patterns

**Figure 1:**
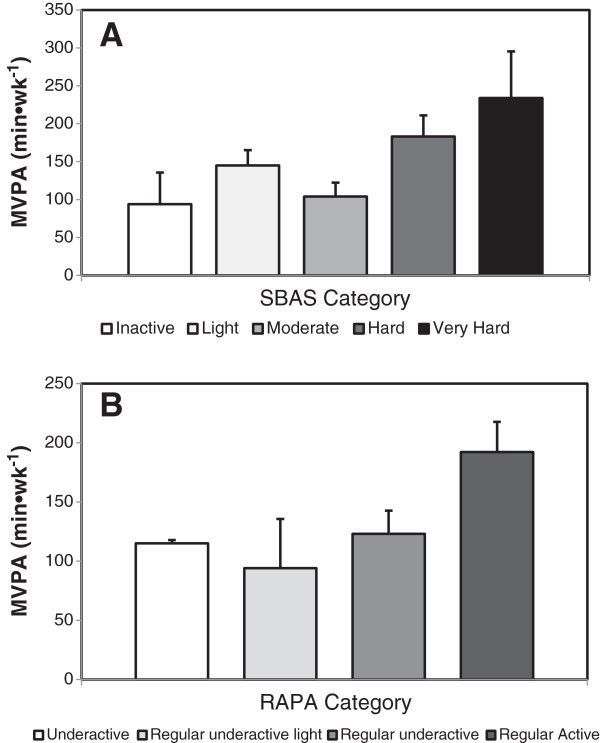
**Objectively measured time spent in moderate and vigorous PA (MVPA) for participants classified in each PA level category as determined by two questionnaires.** According to SBAS categories **(panel A)** participants’ level of PA was classified as inactive (n = 3), light (n = 10), moderate (n = 5), hard (n = 7), and very hard (n = 5). According to RAPA categories **(panel B)**, participants’ level of PA was classified as underactive (n = 2), regular underactive light (n = 3), regular underactive (n = 11), and regular active categories (n = 15). Data shown as mean ± SD and expressed in min•wk^-1^.

Participants’ mean accelerometer wear time was 12.0 ± 2.3 hours•d^-1^. Based on accelerometer assessment of activity mean MVPA was 153 ± 89 min•wk^-1^ (Table 1). Figure 1 illustrates the amount of objectively-measured MVPA for participants that were classified into each survey-assessed PA category. According to SBAS categories, mean MVPA (in min•wk^-1^) for participants classified to the inactive, light, moderate, hard, and very hard categories were 94 ± 74 (n = 3), 145 ± 64 (n = 10), 104 ± 41 (n = 5), 183 ± 74 (n = 7), and 234 ± 137 (n = 5), respectively. According to RAPA categories, mean MVPA (in min•wk^-1^) for participants classified into the underactive, regular underactive light, regular underactive, and regular active categories were 115 ± 4 (n = 2), 94 ± 72 (n = 3), 123 ± 65 (n = 11), and 192 ± 99 (n = 15), respectively.

Fifteen of 31 (48%) of participants met the U.S. PA guidelines of ≥150 min•wk^-1^ of MVPA [41]. Participants classified as meeting the 2008 U.S. PA guidelines based on SBAS PA level spent 42 min•wk^-1^ engaged in MVPA more than those classified as not meeting the guidelines, this difference was not statistically significant (Table 2). When using the RAPA to classify participants based on their compliance with the 2008 U.S. PA guidelines, those who were classified as meeting the guidelines spent significantly more time engaged in MVPA than those who did not meet the guidelines (192 ± 99 min•wk^-1^ of MVPA vs. 117 ± 61 min•wk^-1^ of MVPA, respectively; *p* = 0.018).

**Table 2:** **Time (in min•wk**^**-1**^**) spent engaged in MVPA when participants were classified as meeting or not meeting the 2008 U.S. PA guidelines assessed using accelerometer, the SBAS, and the RAPA**^**1**^

**Assessment method**	**Meeting guidelines**		**Not meeting guidelines**		***p *** **value**^**2**^
	**n**	**Time (min•wk**^**-1**^**)**	**n**	**Time (min•wk**^**-1**^**)**	
Accelerometers	15	221 ± 75	16	90 ± 39	0.0001
SBAS	17	175 ± 100	13	133 ± 66	0.222
RAPA	15	192 ± 99	16	117 ± 61	0.018

^1^Data shown as mean ± SD and expressed in min•wk^-1^; MVPA: Moderate Walking + Vigorous PA.

^2^P-value for independent t-test performed on the square root transformation of MVPA for those meeting and not meeting the 2008 U.S. PA guidelines.

### Survey validation

Validity and test-retest reliability for the SBAS were 0.38 (*p* < 0.05) and 0.61 (*p* < 0.01), respectively (Table 3). The SBAS had sensitivity, specificity, and positive and negative predictive values of 0.60, 0.47, 0.53, and 0.54, respectively.

**Table 3:** Validity, test-retest reliability, sensitivity, specificity, positive and negative predictive values of the Mexican Spanish version of the SBAS and the RAPA compared to accelerometer-measured MVPA

	**SBAS**		**RAPA**	
	**r**	***p *** **value**	**r**	***p *** **value**
Validity^1^	0.38	0.04	0.45	0.01
	**ICC**	***p *** **value**	**ICC**	***p *** **value**
Test-retest reliability^2^	0.61	0.005	0.65	0.002
Sensitivity^3^	0.60		0.73	
Specificity^4^	0.47		0.75	
Positive predictive value^5^	0.53		0.73	
Negative predictive value^6^	0.54		0.75	

^1^Spearman’s rank ordered correlation between MVPA and score on each questionnaire.

^2^Intraclass Correlation Coefficient between the scores on the first and second administration of each questionnaire administered one week apart.

^3^True positives/ (true positives + false negatives).

^4^True negatives/ (true negatives + false positives).

^5^True positives/ all positives.

^6^True negatives/ all negatives.

Validity and test-retest reliability for the RAPA were 0.45 (*p* < 0.05) and 0.65 (*p* < 0.01), respectively. The RAPA had sensitivity, specificity, and positive and negative predictive values of 0.73, 0.75, 0.73, and 0.75, respectively.

## Discussion

The purpose of this work was to explore the utility of the Mexican Spanish version of two questionnaires, the Stanford Brief Activity Survey (SBAS) and the Rapid Assessment of Physical Activity (RAPA), for use as tools to assess PA in Mexican-American adults.

### Validity and test-retest reliability

Both questionnaires showed modest validity compared with *ActiGraph* accelerometer objective measures of PA (r = 0.38 and 0.45 for the SBAS and the RAPA, respectively). Relative to the English version of the SBAS [30], the validity of the Spanish version was lower. One plausible explanation may be the long activity descriptions and lack of cultural relevance in the types of activities given as examples for leisure time and occupational physical activities. This demonstrates the necessity for implementing cultural adaptations when translating physical activity questionnaires to be delivered to the Hispanic population. In the previous validation study of the English version of the SBAS [30,37] participants were aged 60-69, of mixed race, and many had hypertension (60%), hyperlipidemia (45%), metabolic syndrome (26%), or type 2 diabetes (18%). In contrast, participants in the current study were younger and were a more homogenous group as all were Hispanic and none had diagnosis of chronic diseases. This difference in study population may have contributed to the limited distribution of participants among the different categories of PA. In contrast, validity of the Spanish version of the RAPA was of comparable magnitude to that reported for its English version [31]. It must be noted that the studies validating the English version of both questionnaires used a subjective measure of PA for comparison [30,31], whereas the current study used an objective measure of PA. Previous research comparing subjective measures of PA to objective measures reported modest Spearman rank order correlation coefficients of similar magnitude to those reported herein [42]. Moreover, with this approach we were able to demonstrate the concurrent validity of these questionnaires for assessing both, meeting or not meeting the 2008 PA Guidelines [41].

Test-retest reliability was modest when the Spanish version of the surveys was administered in duplicate one week apart (ICC = 0.61 and 0.65 for the SBAS and the RAPA, respectively). Validation studies for the original English version of these surveys did not report test-retest reliability. However, when the English SBAS was administered 2 years apart in participants assigned to the control group of an intervention study; the scores from both assessments were significantly correlated (r = 0.62, *p* < 0.001) [37].

The values that we obtained were comparable to previous validation studies in this population. For example Rauh et al. [43] reported weak to moderate correlations between five self-reported measures of PA and counts obtained using an electronic motion sensor in a sample of Latinos, most of Mexican descent. These surveys also had variable test-retest reliability. In a separate study, the Yale Physical Activity Survey had moderate to good reliability for estimating energy expenditure, and energy spent in select specific activities (shopping, light housework, food preparation) [44]. This suggests that, despite the population age or the type of instrument used, translations into Spanish and completion by Hispanic individuals may involve differences in how the respondents understand the questions as written and intent of the questions asked. However, due to our small sample size (n = 31 RAPA; n = 30 SBAS) the conclusions drawn from these data must be interpreted with caution.

### Sensitivity, specificity, PPV, and NPV

Sensitivity and specificity of the Spanish SBAS were 0.60 and 0.47, respectively. Whereas these values are lower than those determined in the previous validation study with the English version of the SBAS (0.73 and 0.61 for sensitivity and specificity, respectively) in different populations [30], the current study used an objective measure to determine weekly minutes of PA rather than a survey. This indicates the ability of the SBAS to accurately identify those who meet the PA recommendations is better than chance (at 0.50). However no significant differences in minutes of MVPA were observed between those classified as meeting and not meeting the PA guidelines by the SBAS. The low specificity suggests the SBAS may misclassify participants who do not meet the PA guidelines. Furthermore, only 29% of participants in our sample reported not having a job; in the previous validation study with the English SBAS [30] 50% of participants reported no work. This major difference in sample population could also explain why the SBAS did not perform as well with our population, as it did in previous validation studies [30,37]. We selected the SBAS because it has been previously validated and it includes an assessment of occupational PA. Previous research has highlighted the need for capturing this domain of physical activity, as Mexicans engage in occupational PA more frequently than other ethnic groups [22]. However, it is possible that many of the activities included in this survey may lack cultural relevance for Spanish speaking individuals of Mexican descent.

Sensitivity and specificity were 0.73 and 0.75, respectively, for the RAPA. These values are comparable to the validation of the English version of the RAPA in different populations (0.82 and 0.69, respectively) [30,31]. Participants classified as meeting the PA guidelines by the RAPA engaged in 64% more time in MVPA than those who were classified as not meeting the guidelines (p = 0.018). The specificity of the RAPA was slightly higher than the SBAS, indicating better discrimination than the SBAS in correctly classify those who do not meet the 2008 PA guidelines by the questionnaire and accelerometry.

Positive and negative predictive values for both studies were comparable to those values obtained in previous validation studies [30,31]. Positive predictive value and negative predictive value was lower for the SBAS than the RAPA, indicating the RAPA questionnaire is better at discriminating between those who do and do not meet the PA guidelines from the questionnaire. However, an important limitation that must be acknowledged is the relatively small sample size (n = 31, RAPA; n = 30, SBAS) for this type of validation study, which may have reduced the statistical power to detect if the scores from these questionnaires are useful to identify participants who participate in different levels of MVPA, and may have affected the intraclass correlation coefficients used to determine test-retest reliability.

It is noteworthy that participants in the current study had a higher level of MVPA (153.3 ± 38.8 min•wk^-1^) than what has been previously reported in the literature [45]. By study design, we attempted to recruit participants with diverse physical activity habits, which may have led to the inclusion of some participants who were regularly highly active. Nevertheless, only a small proportion of MVPA time was spent in vigorous activity (10.4 ± 24.3 min•wk^-1^) which suggests that the majority of the participants were likely not regular heavy exercisers, but instead engaged in a high level of health enhancing moderate-intensity physical activity.

The use of accelerometers for assessing PA was an important strength of this study, as previous validation studies compared newly developed self-report questionnaires to other self-report measures [30,31]. The current study also has limitations worth acknowledging. The small sample size limited the statistical power and the ability to make any definitive conclusions regarding observed results from this study. Given the heterogeneity within the study sample (wide range in age, time in the US, education, etc.) it is possible that factors not accounted for in this analysis may limit the generalizability of the results reported herein. It is also important to acknowledge that the selected questionnaires are not meant to be used for the assessment of changes in physical activity over time, but rather as a quick screening tool to be used in clinical settings or for the collection of epidemiological data. Finally, although participants were asked to maintain their level of physical activity throughout the duration of the study, the possibility that participants were more active during the week of data collection cannot be ruled out.

## Conclusions

Data from the current study demonstrate the modest ability of the Spanish translated versions of the SBAS and RAPA to predict PA levels. Both questionnaires demonstrated acceptable validity, reliability, specificity, sensitivity, and predictive values against an objective measure of PA. In light of reported discrepancies in PA levels of Mexican Americans based on the type of assessment method used [19,20,33,45] there are recent recommendations to culturally adapt translated surveys to ensure the equivalence and cultural relevance of listed items among the target population [46]. Formative research for the cultural adaptation of these instruments, particularly the SBAS, and inclusion of activities that more closely reflect those in which individuals of Mexican descent engage, such as those of occupational nature [22], would prove beneficial.

## Abbreviations

BMI: Body mass index; Cpm: Counts per minute; ICC: Intraclass correlation coefficients; MVPA: Moderate and vigorous physical activity; NPV: Negative predictive value; PPV: Positive predictive value; RAPA: Rapid Assessment of Physical Activity; SBAS: Stanford Brief Activity Survey; SLML: Sedentary, light, and moderate lifestyle.

## Competing interests

The authors have no financial competing interests to declare.

## Authors’ contributions

SVL was responsible for study design, oversight of data collection and analysis, data interpretation, and manuscript preparation. AC carried out data analysis and contributed to manuscript preparation. KJF participated in study coordination and data collection. BEA assisted with study design, data analysis and interpretation, and manuscript preparation. All authors read and approved the final manuscript.
